# Supplementary material for: The Influence of 5-HTTLPR, BDNF Rs6265 and COMT Rs4680 Polymorphisms on Impulsivity in Bipolar Disorder: The Role of Gender
Source: Genes (Basel). 2022 Mar 9;13(3):482. doi: 10.3390/genes13030482 (PMC8954186; doi:10.3390/genes13030482)
Supplement: Supplementary file 1 [file genes-13-00482-s001.zip › genes-1611899-supplementary/Supplementary Figures.pdf]

# Supplementary Figures

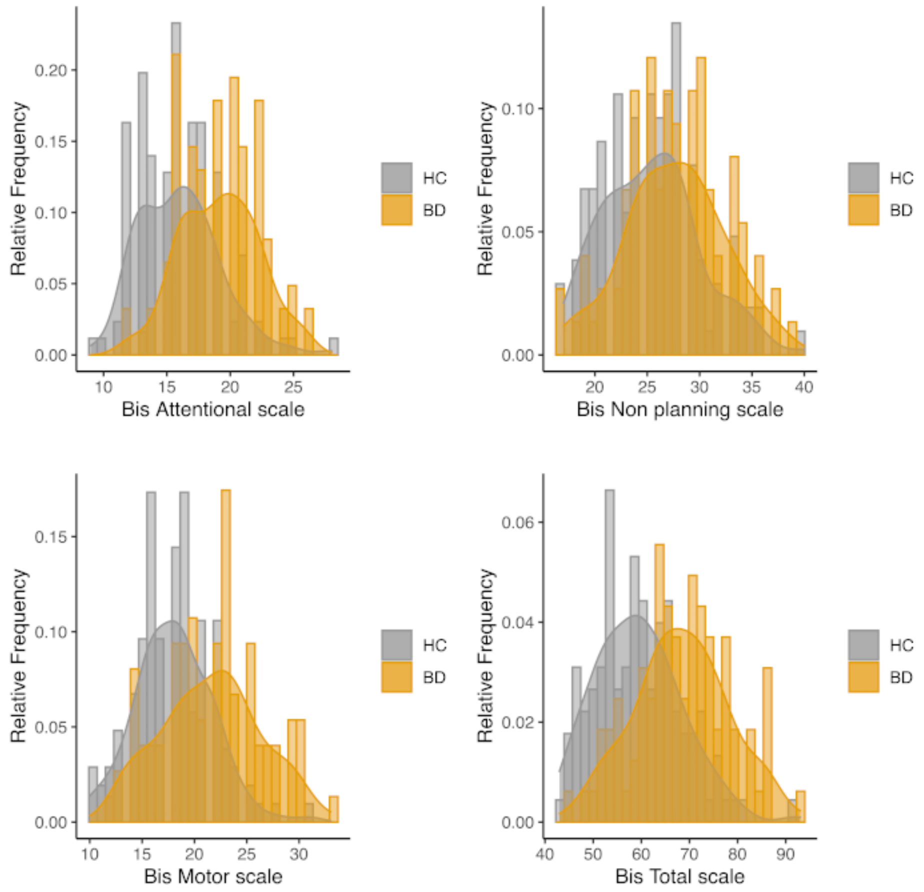


Figure S1: Distribution of BIS-11 score (Attentional, Motor, Non-planning and Total) among HC (Healthy Controls) and BD (Bipolar Disorder

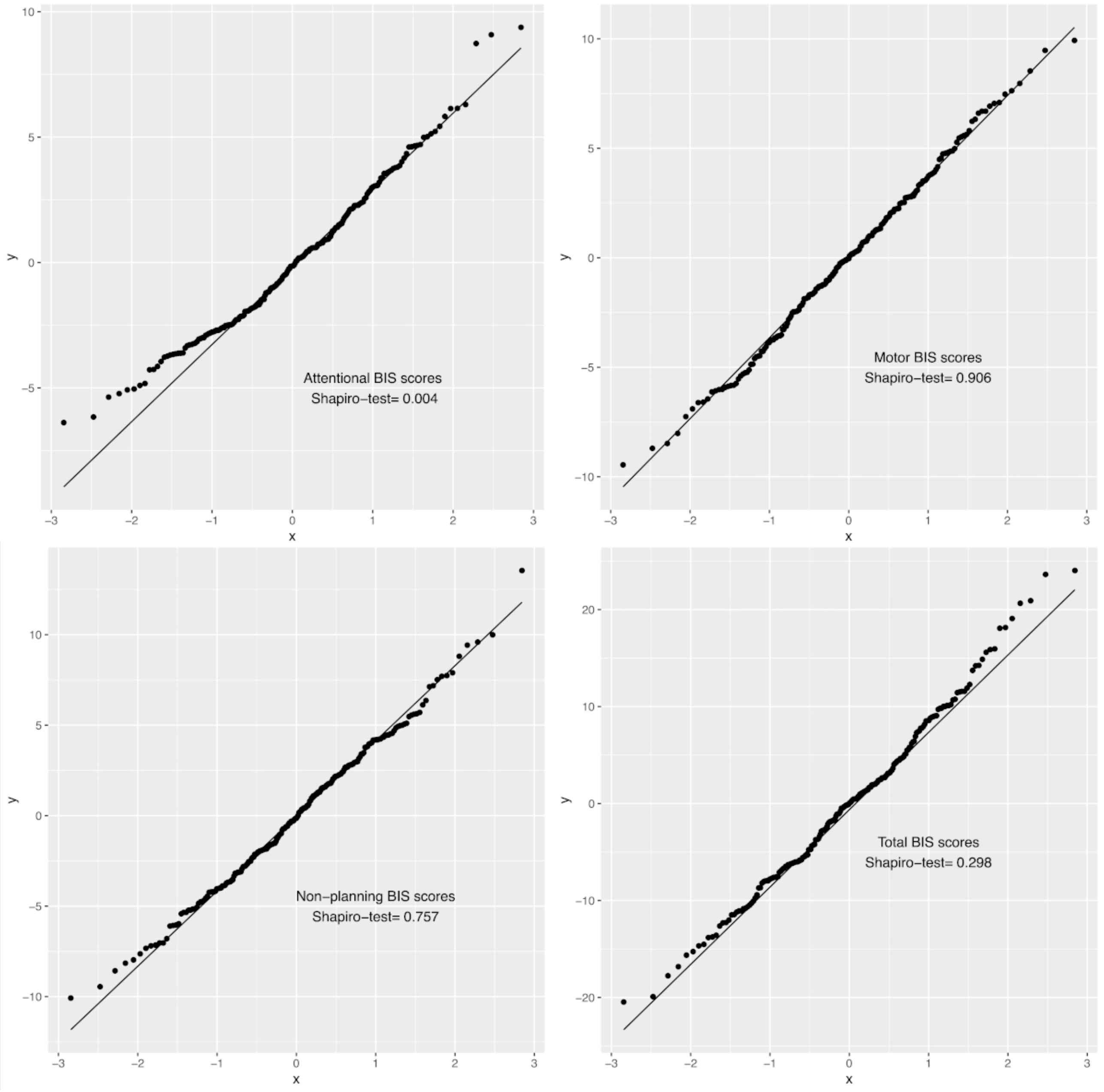


Figure S2. Quantile-quantile diagram for each mixed-effects regression model and p-value of the Shapiro test.
