# Supplementary material for: The Influence of 5-HTTLPR, BDNF Rs6265 and COMT Rs4680 Polymorphisms on Impulsivity in Bipolar Disorder: The Role of Gender
Source: Genes (Basel). 2022 Mar 9;13(3):482. doi: 10.3390/genes13030482 (PMC8954186; doi:10.3390/genes13030482)
Supplement: Supplementary file 1 [file genes-13-00482-s001.zip › genes-1611899-supplementary/TABLE_S1.pdf]

Table S1. Coefficients (β) and relative 95% Interval Confidence estimated by the mixed-effects regression model considering as dependent variable the attentional, motor, non-planning, and total BIS score with respect to the reference category*.

	BIS attentional			BIS motor			BIS non-planning			BIS total		
Predictors	***β***	***95% CI***	***P-value***	***β***	***95% CI***	***P-value***	***β***	***95% CI***	***P-value***	***β***	***95% CI***	***P-value***
(Intercept)	17.7	16.1 – 19.3	**<0.001**	20.3	18.1 – 22.4	**<0.001**	28.7	26.2 – 31.2	**<0.001**	65.7	61.1 – 70.3	**<0.001**
Disease [BD]	1.95	0.92 – 2.98	**<0.001**	2.26	0.92 – 3.60	**0.001**	1.21	-0.29 – 2.70	0.113	5.35	2.40 – 8.30	**<0.001**
Gender [Female]	-0.39	-1.55 – 0.77	0.509	-1.09	-2.62 – 0.44	0.160	-2.42	-4.12 – -0.72	**0.006**	-3.34	-6.67 – -0.01	**0.050**
Age class [31.7-47]	0.47	-0.60 – 1.53	0.389	-0.41	-2.20 – 1.39	0.656	0.99	-0.58 – 2.56	0.214	2.22	-0.84 – 5.27	0.154
Age class [48-73]	0.21	-0.95 – 1.38	0.717	1.01	-0.86 – 2.89	0.288	0.98	-0.72 – 2.67	0.258	2.16	-1.17 – 5.49	0.202
Raven IQ scale [115-127]	-0.63	-1.64 – 0.38	0.222	-1.47	-2.81 – -0.14	**0.030**	-0.45	-1.91 – 1.01	0.545	-2.68	-5.58 – 0.23	0.071
Raven IQ scale [128-128]	-0.52	-1.85 – 0.82	0.446	-0.51	-2.30 – 1.28	0.573	-0.79	-2.72 – 1.15	0.423	-1.80	-5.62 – 2.02	0.355
Educational level [14-18]	-1.10	-2.14 – -0.06	**0.038**	-0.57	-1.94 – 0.79	0.407	-1.25	-3.30 – 0.80	0.232	-2.91	-5.87 – 0.06	0.054
Educational level [19-26]	-1.62	-2.98 – -0.27	**0.019**	-1.15	-2.92 – 0.63	0.204	-2.81	-5.57 – -0.06	**0.045**	-4.00	-7.86 – -0.14	**0.043**
COMT [AA]	-0.81	-1.87 – 0.24	0.132	-1.08	-2.47 – 0.31	0.126	-0.03	-2.36 – 2.30	0.981	-2.29	-5.31 – 0.73	0.136
COMT [GG]	-0.14	-1.08 – 0.80	0.769	-0.39	-1.62 – 0.84	0.534	-1.87	-3.96 – 0.21	0.078	-0.88	-3.57 – 1.82	0.522
X5.HTTLPR [LL]	0.30	-1.09 – 1.68	0.675	-0.40	-2.22 – 1.42	0.665	-2.11	-4.12 – -0.11	**0.039**	-1.59	-5.57 – 2.39	0.431
X5.HTTLPR [SS]	-1.42	-3.25 – 0.42	0.129	-0.25	-2.66 – 2.17	0.841	-2.02	-4.66 – 0.61	0.131	-4.16	-9.42 – 1.10	0.120
BDNF [TC]	0.32	-0.55 – 1.20	0.468	-1.32	-3.23 – 0.59	0.175	0.22	-1.06 – 1.51	0.730	0.86	-1.65 – 3.36	0.501
BDNF [TT]	-1.85	-3.57 – -0.12	**0.036**	-4.00	-7.75 – -0.25	**0.037**	-5.68	-8.17 – -3.19	**<0.001**	-10.2	-15.2 – -5.31	**<0.001**
X5.HTTLPR [LL]*Gender [F]	-0.09	-1.90 – 1.73	0.925	1.53	-0.87 – 3.94	0.210	1.89	-0.77 – 4.55	0.162	2.43	-2.77 – 7.63	0.359
X5.HTTLPR [SS]*Gender [F]	3.82	1.43 – 6.22	**0.002**	4.03	0.89 – 7.17	**0.012**	4.02	0.59 – 7.44	**0.022**	12.0	5.16 – 18.9	**0.001**
BDNF [TC]*Age class [31.7-47]				3.80	1.11 – 6.49	**0.006**						
BDNF [TT]*Age class [31.7-47]				1.24	-4.31 – 6.78	0.660						
BDNF [TC]*Age class [48-73]				0.13	-2.66 – 2.92	0.928						
BDNF [TT]*Age class [48-73]				2.77	-2.58 – 8.11	0.308						
COMT [AA]*Educational level [14-18]							-0.80	-4.18 – 2.59	0.643			
COMT [GG]*Educational level [14-18]							1.24	-1.82 – 4.31	0.425			
COMT [AA]*Educational level [19-26]							-0.60	-4.63 – 3.44	0.771			
COMT [GG]*Educational level [19-26]							5.30	1.60 – 8.99	**0.005**			
σ^2		8.97			15.47			18.22			74.08	
τ_{00}		0.19 $_{centro}$			0.00 $_{centro}$			0.84 $_{centro}$			0.54 $_{centro}$	
ICC		0.02			0.00			0.04			0.01	
N		8 $_{centro}$			8 $_{centro}$			8 $_{centro}$			8 $_{centro}$	
Observations		225			225			225			225	
Marginal R^2/ Conditional R^2		0.281 / 0.296			0.283 / 0.283			0.230 / 0.264			0.331 / 0.336	

** reference category: Disease [HC], Gender [Male], Age-class [<31.7], Raven IQ scale [<115], Educational level [<14], COMT [AG], X5.HTTLPR [LS], BDNF [CC].*
