# Supplementary material for: The Influence of 5-HTTLPR, BDNF Rs6265 and COMT Rs4680 Polymorphisms on Impulsivity in Bipolar Disorder: The Role of Gender
Source: Genes (Basel). 2022 Mar 9;13(3):482. doi: 10.3390/genes13030482 (PMC8954186; doi:10.3390/genes13030482)
Supplement: Supplementary file 1 [file genes-13-00482-s001.zip › genes-1611899-supplementary/TABLE_S2.pdf]

Table S2. Retrospective Design Analysis on the highest observed effect size (Cohen d), the ratio between the observed Standard Deviation (SDratio) and the sample size of each subgroup (n1 and n2) reporting the observed power (P) and the type M (M) and type S (S) errors. In bold the observed power greater than 60%.

	BIS-11 Attentional							BIS-11 Motor							BIS-11 Non-planning							BIS-11 Total						
	d	SDratio	n1	n2	P	M	S	d	SDratio	n1	n2	P	M	S	d	SDratio	n1	n2	P	M	S	d	SDratio	n1	n2	P	M	S
5-HTTLPR * Gender	0.53	1.61	49	20	0.58	1.31	0.00	**0.68**	**0.97**	**32**	**20**	**0.64**	**1.27**	**0.00**	0.31	1.19	64	46	0.37	1.63	0.00	**0.80**	**1.61**	**49**	**20**	**0.91**	**1.07**	**0.00**
BDNF	0.28	0.55	14	80	0.22	2.11	0.01	0.34	0.59	14	131	0.34	1.69	0.01	**0.62**	**0.54**	**14**	**80**	**0.75**	**1.16**	**0.00**	**0.85**	**0.55**	**14**	**80**	**0.95**	**1.04**	**0.00**
COMT	0.10	0.73	50	100	0.10	4.07	0.07	0.11	0.73	50	100	0.11	3.59	0.05	0.18	0.82	75	100	0.21	2.20	0.00	0.16	0.73	50	100	0.18	2.50	0.01
